# Internet Use and Access Among Pregnant Women via Computer and Mobile Phone: Implications for Delivery of Perinatal Care

**DOI:** 10.2196/mhealth.3347

**Published:** 2015-03-30

**Authors:** Rachel Peragallo Urrutia, Alexander A Berger, Amber A Ivins, A Jenna Beckham, John M Thorp Jr, Wanda K Nicholson

**Affiliations:** ^1^Division of Women's Primary HealthcareDepartment of Obstetrics and GynecologyUniversity of North CarolinaChapel Hill, NCUnited States; ^2^Department of Obstetrics and GynecologyJefferson Medical CollegePhiladelphia, PAUnited States; ^3^Center for Women's Health ResearchUniversity of North CarolinaChapel Hill, NCUnited States

**Keywords:** pregnancy, postpartum period, Internet, mobile phone, health behavior, risk reduction behavior

## Abstract

**Background:**

The use of Internet-based behavioral programs may be an efficient, flexible method to enhance prenatal care and improve pregnancy outcomes. There are few data about access to, and use of, the Internet via computers and mobile phones among pregnant women.

**Objective:**

We describe pregnant women’s access to, and use of, computers, mobile phones, and computer technologies (eg, Internet, blogs, chat rooms) in a southern United States population. We describe the willingness of pregnant women to participate in Internet-supported weight-loss interventions delivered via computers or mobile phones.

**Methods:**

We conducted a cross-sectional survey among 100 pregnant women at a tertiary referral center ultrasound clinic in the southeast United States. Data were analyzed using Stata version 10 (StataCorp) and R (R Core Team 2013). Means and frequency procedures were used to describe demographic characteristics, access to computers and mobile phones, and use of specific Internet modalities. Chi-square testing was used to determine whether there were differences in technology access and Internet modality use according to age, race/ethnicity, income, or children in the home. The Fisher’s exact test was used to describe preferences to participate in Internet-based postpartum weight-loss interventions via computer versus mobile phone. Logistic regression was used to determine demographic characteristics associated with these preferences.

**Results:**

The study sample was 61.0% white, 26.0% black, 6.0% Hispanic, and 7.0% Asian with a mean age of 31.0 (SD 5.1). Most participants had access to a computer (89/100, 89.0%) or mobile phone (88/100, 88.0%) for at least 8 hours per week. Access remained high (>74%) across age groups, racial/ethnic groups, income levels, and number of children in the home. Internet/Web (94/100, 94.0%), email (90/100, 90.0%), and Facebook (50/100, 50.0%) were the most commonly used Internet technologies. Women aged less than 30 years were more likely to report use of Twitter and chat rooms compared to women 30 years of age or older. Of the participants, 82.0% (82/100) were fairly willing or very willing to participate in postpartum lifestyle intervention. Of the participants, 83.0% (83/100) were fairly willing or very willing to participate in an Internet intervention delivered via computer, while only 49.0% (49/100) were fairly willing or very willing to do so via mobile phone technology. Older women and women with children tended to be less likely to desire a mobile phone-based program.

**Conclusions:**

There is broad access and use of computer and mobile phone technology among southern US pregnant women with varied demographic characteristics. Pregnant women are willing to participate in Internet-supported perinatal interventions. Our findings can inform the development of computer- and mobile phone-based approaches for the delivery of clinical and educational interventions.

## Introduction

Recent data from the Pew Research Center suggest that access to the Internet is growing rapidly across all segments of the US adult population [1]. Internet-based clinical interventions have grown in popularity [2] and have been used successfully for weight loss [3-5], diabetes management [6,7], physical activity [8,9], and tobacco cessation [10]. There are currently over 61 million women of childbearing age in the United States [11]. The preconception, pregnancy, and postpartum periods are critical teachable moments in the lives of young women, providing opportunities to implement interventions to promote maternal and infant health [12]. As such, Internet-based educational and clinical interventions may be particularly useful to women leading up to, during, and immediately after pregnancy [13].

Pregnant women in Europe report excellent access to Internet-based interventions [14,15]. Pregnant women in the US are thought to have broad access to the Internet, however, there is little published data exploring pregnant women’s access to Internet technologies in the US. Additionally, there is a paucity of data regarding Internet access among pregnant women of different racial/ethnic groups, socioeconomic status, or specific geographical region. Huberty et al, for example, described the use of the Internet by pregnant and postpartum women in a Midwestern US population [16]. However, only Internet-using women were included in the online sampling method, and the population was comprised predominately (70%) of non-Hispanic white women. Because of racial/ethnic differences and the variability of Internet availability within geographical areas, targeted assessment of access to, and use of, specific Internet-based technologies among vulnerable populations, including pregnant women, can assist providers in developing programs to broaden the delivery of health care services. Clinicians, researchers, and policy makers can use this information to develop prenatal and postnatal behavioral interventions that effectively employ Internet-based technologies, tailored to the needs of their target patient populations.

In response to the absence of data on pregnant women, particularly pregnant women living in Central North Carolina, we endeavored to assess women’s access to the Internet using computers and mobile phones and their use of various computer and mobile phone technologies. The prevalence of overweight and obesity among pregnant women at our institutions is about 60%. As such, we also aimed to describe the willingness of pregnant women to participate in an Internet-based, weight-loss intervention delivered via computer or mobile phone.

## Methods

### Study Setting and Sample

We conducted a written, in-person survey among a convenience sample of pregnant women presenting for obstetrical ultrasound at a university-based, tertiary care center in Central North Carolina between March 1, 2011 and May 31, 2011. The ultrasound unit provides care to a racially and socioeconomically diverse group of pregnant women from hospital-based clinics at the university and 14 health departments in the surrounding area. Approximately 300 women per month are referred for ultrasound evaluation. Women were eligible to participate if they were 18 years of age or older and able to provide written informed consent in English. We excluded women presenting for genetic counseling and women with a nonviable pregnancy or a pregnancy complicated by a fetal anomaly. We aimed for a sample size of 100 women based on the assumption of 80% power to detect a 10% difference in our main outcome, which was willingness to participate in a behavioral intervention program via Internet on the computer versus on a mobile phone. The Institutional Review Board of the University of North Carolina approved the study.

### Survey Instrument

The Institute of Medicine access to care model was used as a framework for the study. A questionnaire was developed to examine women’s access to computers and mobile phones according to predisposing (ie, age, race/ethnicity), enabling (ie, income, number of children in the home), and clinical (ie, gestational age) factors [17]. A brief survey was developed following a review of published articles on Internet use among pregnant women. The survey was initially reviewed by 10 pregnant women attending our university-based prenatal clinics. Based on the feedback provided, we made minor edits to the wording of several questions for clarity. The final version consisted of eight items on access to, and use of, computers, mobile phones, and Internet and social media tools, as well as preferences for the delivery of a postpartum weight-loss intervention and demographic information. Each participant was asked the following questions (see Multimedia Appendix 1 for the full survey):

1. “Do you have access to a home phone with Internet? (Yes/No)”

2. “Do you have access to a mobile phone with Internet? (Yes/No)”

3. “Do you have access to a computer for at least 8 hours per week (at home, at work, or in a public setting such as your local library)? (Yes/No)”

4. “How often do you use the following Internet programs (Internet/Web, email, Facebook, blogs, Twitter, chat rooms, Skype) on your computer? (Not at all, Not very often, Often, Very often)”

5. “How often do you use these items (Internet/Web, email, Facebook, blogs, Twitter, chat rooms) on your cell phone? (Not at all, Not very often, Often, Very often)”

6. “How willing are you to participate in a postpartum weight-loss program? (Not at all willing, Not very willing, Fairly willing, Very willing)”

7. “How willing are you to participate in an Internet-based postpartum weight-loss program delivered via the computer? (Not at all willing, Not very willing, Fairly willing, Very willing)”

8. “How willing are you to participate in an Internet-based postpartum weight-loss program on your cell phone? (Not at all willing, Not very willing, Fairly willing, Very willing)”

### Procedure

Women were approached consecutively by trained research staff to ascertain their interest in participating in the study as they presented for their ultrasound appointment. If women expressed an interest, they were escorted to a private conference room within the ultrasound unit where they were screened for eligibility. If they were deemed eligible, informed consent was obtained and they were officially enrolled in the study. The average time to complete the survey was 10 minutes. The research staff reviewed each survey for completeness in the presence of the participant. Participants who completed the survey received a US $10 gift card. Data were entered into a master spreadsheet by a study staff member. As part of our quality control protocol, a second staff member conducted an audit of the data each month to ensure correct entries. There were no missing data.

### Data Analysis

Maternal demographic and clinical characteristics were summarized using means and standard deviations for continuous variables and numbers with proportions for categorical variables. The proportion of participants with access to computers and mobile phones across predisposing (ie, age, race/ethnicity), enabling (ie, income, number of children in the home), and clinical (ie, gestational age) factors was assessed using chi-square statistics. Given the performance of four tests and following the Bonferroni correction for multiple testing [18], results were considered statistically significant if *P*<.013 (.05 divided by three). For analyses stratified by race/ethnicity, we recategorized African American, Asian, and Hispanic women into one category, termed *nonwhite*, due to small numbers.

Willingness to participate in the various types of interventions was defined as those who answered *fairly willing* or *very willing* and was compared to the number who answered *not at all willing* or *not very willing*. Willingness to participate in a postpartum weight-loss intervention across sociodemographic groups was assessed using Fisher’s exact test. The *P* values were corrected for multiple testing with a Benjamini-Hochberg correction [19]. To determine which demographic characteristics were associated with women’s willingness to participate in computer-based or mobile phone-based weight-loss interventions, we first performed univariate logistic regression. The models looked at willingness to participate in interventions via computer and mobile phone according to age (continuous), race/ethnicity (nonwhite versus white), income level (≥US $25,000 versus <US $25,000), and number of children in the home (one or more versus none). We then included all the above variables in a multiple logistic regression model to determine if any were predictive of increased willingness to participate in an Internet intervention by computer or by mobile phone. Again, based on a Bonferroni correction [18], results were considered statistically significant if the *P* value was <.006. Analyses were conducted using Stata version 10 statistical software (StataCorp) and R (R Core Team 2013).

## Results

### Overview

Of the 120 women approached, 110 (91.7%) were eligible for the study and 100 (83.3%) agreed to participate. Of those approached, 10 out of 120 (8.3%) women were not eligible to participate in the study due to a nonviable pregnancy (9/120, 7.5%) or pregnancy complicated by a fetal anomaly (1/120, 0.8%). Of those approached, 10 out of 120 (8.3%) women declined to participate due to a lack of time to complete the survey. The study sample represents approximately 10% of the patients presenting to the ultrasound unit during the 3-month study period.

### Characteristics of the Study Sample

The mean age of participants was 31.0 (SD 5.1) years (see Table 1) with a range of 22 to 44 years. Of the participants, 61.0% (61/100) were white, 26.0% (26/100) were African American, 7.0% (7/100) were Asian, and 6.0% (6/100) were Hispanic. Of the participants, 45.0% (45/100) reported a yearly household income of more than US $50,000. Median gestational age at the time of the survey was 26.0 weeks ± 9.2 with a range of 7 to 41 weeks. Most participants (64/100, 64.0%) had one or more children in the home. See Table 1 for further demographic information.

**Table 1:** Selected sociodemographic and clinical characteristics of study participants (n=100).

Characteristics		mean (SD), n (%), or median (range)
Age in years, mean (SD)		31.0 (5.1)
**Race/ethnicity, n (%)**		
	White	61 (61.0)
	African American	26 (26.0)
	Asian	7 (7.0)
	Hispanic	6 (6.0)
**Yearly household income, n (%)**		
	≤US $25,000	28 (28.0)
	US $25,001-$50,000	27 (27.0)
	>US $50,000	45 (45.0)
Number of children in the home, median (range)		1.2 (0-5)
Gestational age in weeks, median (range)		26.0 (7-41)

### Computer and Mobile Phone Internet Access

Most participants reported access to computers (89/100, 89.0%) and mobile phones (88/100, 88.0%). White women were more likely to report access to both computers and mobile phones compared to nonwhite women (*P*=.007, see Table 2). There were no statistically significant differences in access to computers or mobile phones by age.

Women with one or more children in the home reported slightly less access to both mobile phones and computers than those with no children in the home, but this difference was not statistically significant (*P*=.07)—94.0% (94/100) versus 81.0% (81/100), respectively (see Table 3). There were no statistically significant differences in access to computers or mobile phones by income or by number of children in the home.

**Table 2:** Self-reported computer and mobile phone Internet access among pregnant women stratified by age and race (n=100).

Internet variables	Total (n=100)	Age in years		*P* value	Race		*P* value
		<30 (n=37)	≥30 (n=63)		White (n=61)	Nonwhite (n=39)	
Access to computer with Internet, n (%)	89 (89.0)	32 (86)	57 (90)	.5	57 (93)	32 (82)	.04
Access to mobile phone with Internet, n (%)	88 (88.0)	33 (89)	55 (87)	.8	57 (93)	31 (79)	.08
Access to both computer and mobile phone, n (%)	86 (86.0)	32 (86)	54 (86)	.9	57 (93)	29 (74)	.007

**Table 3:** Self-reported computer and mobile phone Internet access among pregnant women stratified by income and number of children in the home.

Internet variables	Total (n=100)	Annual income			*P* value	Number children in the home		*P* value
		≤US $25,000 (n=28)	US $25,000-$50,000 (n=27)	>US $50,000 (n=45)		None (n=36)	One or more (n=64)	
Access to computer with Internet, n (%)	89 (89.0)	25 (89)	24 (89)	40 (89)	.998	34 (94)	55 (86)	.20
Access to mobile phone with Internet, n (%)	88 (88.0)	24 (86)	25 (93)	39 (87)	.70	34 (94)	54 (84)	.15
Access to both computer and mobile phone, n (%)	86 (86.0)	23 (82)	24 (89)	39 (87)	.80	34 (94)	52 (81)	.07

### Willingness to Participate in an Online Postpartum Weight-Loss Intervention

Of the women surveyed, 82.0% (82/100) were *very willing* or *fairly willing* to participate in an online weight-loss intervention program after delivery. Bivariate analysis did not show any statistically significant differences in the willingness versus nonwillingness to participate in an online intervention by age, race, or income categories. Women with no children in the home were more likely to be *very willing* or *fairly willing* to participate in a postpartum program compared to women with one or more children in the home (*P*=.07)—93.0% (93/100) versus 77.0% (77/100), respectively. When asked whether they were willing to participate in a postpartum Internet-based intervention delivered via mobile phone or computer, women were significantly more willing to participate via computer (83/100, 83.0%) compared to by mobile phone (49/100, 49.0%) (*P*<.001).

### Willingness to Engage in an Online Intervention via Computer or Mobile Phone

To explore the individual contributions of demographic factors on willingness to participate in a computer-based or mobile phone-based intervention, we developed separate logistic regression models for each modality (Table 4). In both bivariate and adjusted analyses, there were no statistically significant findings. Women of different ages, races, income levels, and number of children at home were similarly willing to participate in computer-based or mobile phone-based interventions.

**Table 4:** Association of demographic characteristics of pregnant women *very willing* and *fairly willing* to participate in an online weight-loss intervention delivered via computer or mobile phone.

Variables		Computer-based intervention					Mobile phone-based intervention				
		n (%)	Regression coefficient (log OR^a^)	Crude OR (95% CI)	Adjusted OR (95% CI)	*P* value	n (%)	Regression coefficient (log OR)	Crude OR (95% CI)	Adjusted OR (95% CI)	*P* value
**Age range in years** ^**b,c**^ **, n (%)**											
	21-25 (n=16)	14 (88)	1.5	1.0 (0.9-1.1)	1.0 (0.9-1.1)	.86	14 (88)	2.4	0.9 (0.9-1.0)	0.9 (0.8-1.0)	.08
	26-30 (n=34)	28 (82)					14 (41)				
	31-35 (n=27)	20 (74)					10 (37)				
	36-40 (n=21)	20 (95)					10 (48)				
	41-45 (n=2)	1 (50)					1 (50)				
**Race/ethnicity, n (%)**											
	White (n=61)	49 (80)	Ref^d^	Ref	Ref		29 (48)	Ref	Ref	Ref	
	Nonwhite (n=39)	34 (87)	1.9	0.6 (0.2-1.8)	0.6 (0.2-1.9)	.35	20 (51)	0.1	0.9 (0.4-1.9)	0.7 (0.3-1.8)	.48
**Income, n (%)**											
	<US $25,000 (n=28)	22 (79)	Ref	Ref	Ref		13 (46)	Ref	Ref	Ref	
	≥US $25,000 (n=72)	61 (85)	1.6	1.0 (0.5-1.8)	1.0 (0.5-2.2)	.91	36 (50)	-0.1	1.0 (0.7-1.7)	1.3 (0.7-2.3)	.44
**Children at home, n (%)**											
	None (n=36)	31 (86)	Ref	Ref	Ref		23 (64)	Ref	Ref	Ref	
	One or more (n=64)	52 (81)	1.8	0.7 (0.2-2.2)	0.7 (0.2-2.2)	.49	26 (41)	0.6	0.4 (0.2-0.9)	0.4 (0.2-1.1)	.07

^a^odds ratio (OD).

^b^Reference variable is 21 years old.

^c^Age is a continuous variable but selected intervals are presented here.

^d^Reference (Ref) variable.

### Use of Internet Technologies

The majority of participants reported that they *often*, or *very often*, used the Internet (94/100, 94.0%), email (90/100, 90.0%), and Facebook (59/100, 59.0%) through a computer. While the use of specific technologies among sociodemographic groups was not significantly different, there were several important trends noted. In an analysis stratified by age, women under 30 years of age were more likely to report the use of Twitter and chat rooms compared to women 30 years and older (Table 5). Nonwhite women were more likely to report using a computer to access Facebook (26/39, 67%) or chat rooms (5/39, 13%) compared to their white counterparts—54% (33/61) and 5% (3/61), respectively. While the Internet/Web, email, and Facebook were commonly accessed through computers, a smaller proportion of women reported they *often* or *very often* used these technologies via mobile phone—49.0% (49/100), 43.0% (43/100), and 36.0% (36/100), respectively. Women aged 30 and older were less likely to report the use of Facebook *often* or *very often* via mobile phone compared to women under age 30.

**Table 5:** Number of pregnant women reporting the use of Internet technologies as *very often* and *often* on computers or mobile phones by age and race.

Internet variables		Age in years		Race		Total (n=100)
		<30 (n=37)	≥30 (n=63)	White (n=61)	Nonwhite (n=39)	
**Internet program used** *very often* **or** *often* **with computers, n (%)**						
	Internet/Web	34 (92)	60 (95)	59 (97)	35 (90)	94 (94.0)
	Email	31 (84)	59 (94)	56 (92)	34 (87)	90 (90.0)
	Facebook	22 (60)	37 (59)	33(54)	26 (67)	59 (59.0)
	Blogs	9 (24)	14 (22)	17 (28)	6 (15)	23 (23.0)
	Twitter	6 (16)	5 (8)	7 (12)	4 (10)	11 (11.0)
	Chat rooms	4 (11)	4 (6)	3 (5)	5 (13)	8 (8.0)
	Skype	2 (5)	10 (16)	7 (12)	5 (13)	12 (12.0)
**Internet program used** *very often* **or** *often* **with mobile phones, n (%)**						
	Internet/Web	20 (54)	29 (46)	28 (46)	21 (54)	49 (49.0)
	Email	14 (38)	29 (46)	26 (43)	17 (44)	43 (23.0)
	Facebook	18 (49)	18 (29)	17 (28)	19 (49)	36 (36.0)
	Blogs	5 (14)	1 (2)	3 (5)	3 (8)	6 (6.0)
	Twitter	4 (11)	4 (6)	5 (8)	3 (8)	8 (8.0)
	Chat rooms	1 (3)	1 (2)	1 (2)	1 (3)	2 (2.0)

Women with one or more children in the home were less likely to report the use of the Internet via computer compared to women with no children in the home. Women with one or more children in the home reported less use of a mobile phone to access email, Facebook, and other technologies compared to women with no children in the home. The lowest-income women were less likely to access most Internet technologies via computer and mobile phone than women in higher-income levels, as seen in Table 6.

**Table 6:** Number of pregnant women reporting the use of Internet technologies *very often* or *often* on computers or mobile phones by income and number of children in the home.

Internet variables		Annual household income			Number children in the home		Total (n=100)
		<US $25,000 (n=28)	US S25,000-$50,000 (n=27)	>US $50,000 (n=45)	None (n=36)	One or more (n=64)	
**Internet program used** *very often* **or** *often* **with computers, n (%)**							
	Internet/Web	24 (86)	26 (96)	44 (98)	59 (97)	35 (88)	94 (94.0)
	Email	23 (82)	24 (89)	43 (96)	56 (92)	34 (87)	90 (90.0)
	Facebook	17 (60)	18 (67)	24 (53)	33 (54)	26 (67)	59 (59.0)
	Blogs	3 (11)	9 (33)	11 (24)	17 (28)	6 (15)	23 (23.0)
	Twitter	2 (7)	4 (15)	5 (11)	7 (12)	4 (10)	11 (11.0)
	Chat rooms	4 (14)	3 (11)	1 (2)	3 (5)	5 (13)	8 (8.0)
	Skype	1 (4)	7(2)	9 (20)	7 (12)	5 (13)	12 (12.0)
**Internet program used** *very often* **or** *often* **with mobile phones, n (%)**							
	Internet/Web	12 (43)	15 (56)	22 (49)	24 (67)	21 (54)	49 (49.0)
	Email	8 (29)	11 (41)	24 (53)	18 (50)	17 (44)	43 (43.0)
	Facebook	10 (36)	12 (44)	14 (31)	16 (44)	19 (49)	36 (36.0)
	Blogs	1 (4)	1 (4)	4 (9)	11 (4)	3 (8)	6 (6.0)
	Twitter	2 (7)	3 (11)	3 (7)	5 (14)	3 (8)	8 (8.0)
	Chat rooms	1 (4)	0 (0)	1 (2)	1 (3)	1 (3)	2 (2.0)

## Discussion

### Principal Findings

Investigations among nonpregnant, adult populations suggest that behavioral interventions delivered through computers and mobile phones are effective [4-10]. Some groups of pregnant women frequently access health information via the Internet and may do so in lieu of conversations with health care providers [16,20]. Text4Baby is an example of a mobile phone educational campaign accessed by thousands of pregnant women across the US [21,22]. At least one small trial of an Internet-based intervention with pregnant women improved compliance with medical therapy and physical activity [9]. Health care providers and researchers are interested in scalable computer-based or mHealth interventions for larger populations of pregnant women, but more information about access to, and use of, Internet technologies among pregnant women is needed.

Pregnant women in our study reported broad access to the Internet through computers and mobile phones and use of a variety of Internet technologies, including Internet/Web browsers, email, and Facebook. Although nonwhite women and women with one or more children in the home may have less access to the Internet than white women with no children at home, access to, and use of, the Internet was quite high across all groups. Less access by nonwhite women and women with children in the home may be due, in part, to financial restraints or competing demands on time [23].

The majority of women in the study sample were willing to participate in an Internet-based behavioral intervention via computer (83/100, 83.0%), but not as willing to participate via mobile phone (49/100, 49.0%). A recent US study found that 50% of the pregnant women surveyed used the Internet to access health information. However, the survey was administered online and may not be generalizable to larger groups of women [16]. Although other studies have found that pregnant women use the Internet to access pregnancy and childbirth-related information, guidance on physical activity, and newborn health, the data are largely limited to Caucasian women and international populations [14,15,24,25]. It may be that women use mobile phones to access specific screening or treatment information, but may not be as willing to use mobile phones for ongoing weight-management interventions. We did not ascertain the extent to which women used computers or mobile phones to gather health information in the current study. Future studies that examine women’s access and use of the Internet will need to explore what types of information women are searching for when using computers or mobile phones.

In our study, willingness to participate in a mobile phone intervention was not dependent on age, race, income level, or having a child at home. Some trends were noted which may be important to investigate in a larger sample. Younger women and women with no children at home tended to be more willing to participate in a mobile phone-based intervention. Our findings support the development of Internet-based clinical and educational interventions for expectant mothers. Developing mHealth programs or interventions that can be delivered through computer-based technologies may promote better uptake based on individual patient preferences. For example, only 8% of Dutch pregnant women used an email-based intervention [26]. Additionally, further work is needed to better understand the influence of other children in the home on less access to the Internet and, possibly, lower willingness to engage in computer- or mobile phone-based interventions.

The broad access and use of the Internet by women in our sample is similar to rates reported by a 2011 Pew Research Center report [27,28]. In the Pew report, minority women reported slightly lower access to the Internet compared to their white women counterparts (73% versus 94%, respectively). Our findings differ from the Pew study in that we did not find substantially less access to the Internet among lower-income women compared to higher-income women. This difference may be due to the younger age range of our sample of study participants compared with the national sample used in the Pew study. Our findings suggest less of a “digital divide” based on income levels in younger, reproductive-age women. These findings further emphasize the need for targeted studies to better describe access to, and use of, the Internet and other computer technologies in different patient groups (eg, pregnant women) and geographical areas (eg, Central North Carolina).

### Limitations

Our study has several limitations that deserve attention. First, our descriptive study includes a relatively small sample size. Therefore, there is limited power to detect differences between sociodemographic groups. We found some small differences in access between white and nonwhite women and also between women with and without children at home. The survey was investigator developed and, therefore, had not been extensively tested prior to its use. Our data also relies on self-reporting. However, given the nature and setting of the survey, it is unlikely that women were biased in their responses. Also, we did not provide specific information on the weight-loss intervention, so participants may have responded differently if they had been given additional information. Another limitation is that our survey does not explore women’s health information-seeking behavior on the Internet. Access to Internet technologies and use of such technologies is not the same as using such technologies for health applications. For example, in a recent study of Internet users in the US, adults of lower socioeconomic status were less likely to use the Internet for health information seeking specifically [29]. However, use of the Internet specifically for health information seeking is not a prerequisite to participating in an Internet-based behavioral intervention. It is also not a known indicator of uptake of such interventions.

Because our study was conducted in one tertiary care center, it can be argued that the findings have limited generalizability. However, we recruited women referred for ultrasound examination from a wide range of community settings and economic backgrounds. Nevertheless, these findings should be confirmed in a larger sample of women living in a diverse community and receiving care in multiple tertiary care settings.

### Conclusions

This study represents a first step in characterizing access to the Internet among a diverse group of pregnant women in the United States. Larger scale studies across geographical regions are needed to better inform and tailor the development of Web-based interventions to take advantage of this critical period in a woman’s lifespan. Integrating Web-based educational interventions into pregnancy and the postpartum periods may lead to improved maternal and newborn outcomes through improved access and education. Such interventions could change the current paradigm of perinatal care, giving clinicians an opportunity to provide ongoing care to pregnant women between prenatal visits [9]. Internet-based interventions to improve clinical care of pregnant women should also be considered. For example, Internet-based interventions might include feedback and communication between patients and providers about a variety of prenatal disease conditions, screening tests, and shared decision making. Our findings suggest broad access to the Internet through computers and mobile phones for pregnant women across multiple demographic strata. Also, our findings suggest that interventions designed to be delivered using contemporary technologies should be accessible via computers or mobile phones and able to be scaled to meet the needs and preferences of women based on age, race, and the presence of other children in the home that may influence access and sustainable use of the Internet.

## Multimedia Appendix 1

Patient questionnaire.

## Abbreviations

OD: odds ratio

## References

[1] Rainie L (2010). Pew Research Center.

[2] Tate DF, Wing RR, Winett RA (2001). Using Internet technology to deliver a behavioral weight loss program. JAMA.

[3] Tate DF, Jackvony EH, Wing RR (2003). Effects of Internet behavioral counseling on weight loss in adults at risk for type 2 diabetes: a randomized trial. JAMA.

[4] Tate DF, Jackvony EH, Wing RR (2006). A randomized trial comparing human e-mail counseling, computer-automated tailored counseling, and no counseling in an Internet weight loss program. Arch Intern Med.

[5] Appel LJ, Clark JM, Yeh H-C, Wang N-Y, Coughlin JW, Daumit G, Miller ER, Dalcin A, Jerome GJ, Geller S, Noronha G, Pozefsky T, Charleston J, Reynolds JB, Durkin N, Rubin RR, Louis TA, Brancati FL (2011). Comparative effectiveness of weight-loss interventions in clinical practice. N Engl J Med.

[6] Glasgow RE, Kurz D, King D, Dickman JM, Faber AJ, Halterman E, Woolley T, Toobert DJ, Strycker LA, Estabrooks PA, Osuna D, Ritzwoller D (2012). Twelve-month outcomes of an Internet-based diabetes self-management support program. Patient Educ Couns.

[7] Richardson CR, Mehari KS, McIntyre LG, Janney AW, Fortlage LA, Sen A, Strecher VJ, Piette JD (2007). A randomized trial comparing structured and lifestyle goals in an internet-mediated walking program for people with type 2 diabetes. Int J Behav Nutr Phys Act.

[8] Marcus BH, Lewis BA, Williams DM, Dunsiger S, Jakicic JM, Whiteley JA, Albrecht AE, Napolitano MA, Bock BC, Tate DF, Sciamanna CN, Parisi AF (2007). A comparison of Internet and print-based physical activity interventions. Arch Intern Med.

[9] Kim C, Draska M, Hess ML, Wilson EJ, Richardson CR (2012). A web-based pedometer programme in women with a recent history of gestational diabetes. Diabet Med.

[10] Bock BC, Graham AL, Whiteley JA, Stoddard JL (2008). A review of web-assisted tobacco interventions (WATIs). J Med Internet Res.

[11] (2011). US Department of Health and Human Services, Health Resources and Services Administration, Maternal and Child Health Bureau.

[12] McBride CM, Emmons KM, Lipkus IM (2003). Understanding the potential of teachable moments: the case of smoking cessation. Health Educ Res.

[13] Gold KJ, Boggs ME, Mugisha E, Palladino CL (2012). Internet message boards for pregnancy loss: who's on-line and why?. Womens Health Issues.

[14] Larsson M (2009). A descriptive study of the use of the Internet by women seeking pregnancy-related information. Midwifery.

[15] Leune A-S, Nizard J (2012). [Doctor Google: use of Internet during pregnancy in France in 2009]. J Gynecol Obstet Biol Reprod (Paris).

[16] Huberty J, Dinkel D, Beets MW, Coleman J (2013). Describing the use of the internet for health, physical activity, and nutrition information in pregnant women. Matern Child Health J.

[17] Millman M, Committee on Monitoring Access to Personal Health Care Services, Institute of Medicine (1993). Access to Health Care in America.

[18] Dunn OJ (1961). Multiple comparisons among means. J Am Stat Assoc.

[19] Hochberg Y, Benjamini Y (1990). More powerful procedures for multiple significance testing. Stat Med.

[20] Kraschnewski JL, Chuang CH, Poole ES, Peyton T, Blubaugh I, Pauli J, Feher A, Reddy M (2014). Paging "Dr. Google": does technology fill the gap created by the prenatal care visit structure? Qualitative focus group study with pregnant women. J Med Internet Res.

[21] Whittaker R, Matoff-Stepp S, Meehan J, Kendrick J, Jordan E, Stange P, Cash A, Meyer P, Baitty J, Johnson P, Ratzan S, Rhee K (2012). Text4baby: development and implementation of a national text messaging health information service. Am J Public Health.

[22] Evans WD, Abroms LC, Poropatich R, Nielsen PE, Wallace JL (2012). Mobile health evaluation methods: the Text4baby case study. J Health Commun.

[23] Setse R, Grogan R, Cooper LA, Strobino D, Powe NR, Nicholson W (2008). Weight loss programs for urban-based, postpartum African-American women: perceived barriers and preferred components. Matern Child Health J.

[24] Lagan BM, Sinclair M, Kernohan WG (2011). What is the impact of the Internet on decision-making in pregnancy? A global study. Birth.

[25] Gao LL, Larsson M, Luo SY (2013). Internet use by Chinese women seeking pregnancy-related information. Midwifery.

[26] Bot M, Milder IE, Bemelmans WJ (2009). Nationwide implementation of Hello World: a Dutch email-based health promotion program for pregnant women. J Med Internet Res.

[27] Smith A (2012). Pew Research Center.

[28] Zickuhr K, Smith A (2012). Pew Research Center.

[29] Kontos E, Blake KD, Chou WS, Prestin A (2014). Predictors of eHealth usage: insights on the digital divide from the Health Information National Trends Survey 2012. J Med Internet Res.

