# Supplementary material for: Internet Use and Access Among Pregnant Women via Computer and Mobile Phone: Implications for Delivery of Perinatal Care
Source: JMIR Mhealth Uhealth. 2015 Mar 30;3(1):e25. doi: 10.2196/mhealth.3347 (PMC4395770; doi:10.2196/mhealth.3347)
Supplement: Supplementary file 1 [file mhealth_v3i1e25_app1.pdf]

Women's Use of Computer and Mobile Phone Technology in Pregnancy and Postpartum

Purpose: To assess pregnant and postpartum women's use of different computer/cell phone programs as well as their interest in a web-based postpartum weight loss program.

Please answer each of the following questions completely. Your answers are confidential.

Please answer the questions below about your access to computers and cell phones

1. Do you have access to a computer with internet? Yes No

2. Do you have access to a mobile phone with internet? Yes No

3. Do you have access to a computer for at least 8 hours per week (at home, at work or in a public setting, such as your local library)? Yes No

---

The following questions ask about your experiences using different types of programs on your computer or cell phone.

4. How often do you use the following programs on your computer?

   (1= not at all, 2= Not very often, 3=Often, 4= Very often)

a. Internet/Web	1	2	3	4
b. Blogs	1	2	3	4
c. Email	1	2	3	4
d. Chat rooms	1	2	3	4
e. Skype	1	2	3	4

f.	Twitter	1	2	3	4
g.	Facebook	1	2	3	4

5. How often do you use the following programs on your mobile phone?

   (1= not at all, 2= Not very often, 3=Often, 4= Very often)

a. Internet/Web	1	2	3	4
b. Blogs	1	2	3	4
c. Email	1	2	3	4
d. Chat rooms	1	2	3	4
e. Twitter	1	2	3	4
f. Facebook	1	2	3	4

We would like to ask you about <u>your willingness</u> to participate in a postpartum weight loss program. (1= not at all, 2= not very willing, 3=fairly willing, 4= very willing)

6.	How willing are you to participate in a weight loss research program for women after delivery?	1	2	3	4

If the response to Question 4 is "not at all," please go to Question 9; otherwise, please go to Question 5.

7.	How willing are you to participate in a weight loss Program delivered through the Internet/web on your computer?	1	2	3	4

8. How willing are you to participate in a postpartum healthy lifestyle program delivered through the internet/web on your mobile phone? 1 2 3 4

Please answer the following questions about yourself.

9. What is your age? ___ (yrs.)

10. How many children do you have in the home (not including this pregnancy)? _______

11. How would you classify yourself? (Check all that apply)
    1Non-Hispanic white/ Caucasian
    2Non-Hispanic African American
    3Asian
    4Other (please specify):_________

12. What is your average household income?
    1$7,500 or less
    2$7,50l to 15,000
    3$15,001 to 25,000
    4$25,001 to 35,000
    5$35,001 to 50,000
    6 $50,000 or more

THANK YOU FOR COMPLETING THIS SURVEY!
